# Fermented soybean meal using *Bacillus subtilis* and *Aspergillus oryzae* positively enhances cecal microbial composition and broiler performance

**DOI:** 10.5713/ab.250400

**Published:** 2025-11-14

**Authors:** Novi Akhirini, Wara Pratitis Sabar Suprayogi, Pramita Nindya Saraswati, Adi Ratriyanto, Agung Irawan

**Affiliations:** 1 Vocational Program of Animal Husbandry, Vocational School, Universitas Sebelas Maret, Surakarta, Indonesia; 2 Department of Animal Science, Faculty of Animal Science, Universitas Sebelas Maret, Surakarta, Indonesia

**Keywords:** Antinutritional Factor, Microbiome, Poultry, Solid-state Fermentation

## Abstract

**Objective:**

This study investigated the effect of fermented soybean meal (FSBM) prepared through solid-state fermentation using *Bacillus subtilis* (BS) and *Aspergillus oryzae* (AO) to replace SBM in broiler chickens’ diets on production, digestibility, and cecal microbial profile.

**Methods:**

In total, 160 sex-mixed day-old chicks of Cobb 500 broiler chickens were randomly assigned to four groups, four pens (replicates; 10 birds each pen), and were raised for 35 days under tropical conditions. The treatments were control (basal diet; CON) or SBM replaced by FSBM produced using AO (AO group), BS (BS group), and their combination (AO+BS group).

**Results:**

Birds fed the AO+BS diet resulted in higher (p = 0.003) body weight (BW) while the BS diet tended (p = 0.063) to have higher final BW than CON or AO. Similarly, birds fed FSBM prepared under either BS or AO+BS had higher feed intake (p<0.01) than the CON group. No difference was found in feed conversion ratio. Relative organ weights including heart, liver, abdominal fat, and total inner organs were lower (p<0.01) in birds fed the AO+BS diet than the CON group, but relative carcass weight was unaffected. Treatments with BS or AO+BS increased dry matter (DM; p = 0.032), organic matter (p = 0.016), and crude protein (CP; p = 0.044) digestibility, while AO did not affect DM and CP digestibility. Broilers fed the AO+BS diet showed greater abundance of Firmicutes phylum and *Bacteroides* genus than the CON group. Several microbial taxa biomarkers were identified via linear discriminant analysis effect size analysis, including higher abundance of *Enterococcus* and *Bacillus* in the AO+BS group but lower abundance of *Erysipelatoclostridium*, *Odoribacter*, *Ruminococcaceae bacterium*, *Staphylococcus*, and *Clostridium methylpentosum* group in the CON group.

**Conclusion:**

*B. subtilis* and *A. oryzae* could synergistically enhance the nutritional quality of SBM, positively alter cecal microbiota, and improve the production performance of broiler chickens.

## INTRODUCTION

The global broiler industry faces increasing pressure to improve production efficiency to meet consumers’ demand while lowering its environmental impact. In the feed formulation, soybean meal (SBM) remains the primary source of amino acids (AAs), accounting for up to 30% of diets. However, its excellent nutritional composition is often compromised by antinutritional factors (ANF) such as phytic acid, trypsin inhibitors, and lectins, which can inhibit the nutrient absorption, thereby lowering nutrient utilization efficiency [1,2]. In addition, the allergenic properties of protein in SBM are also a concern for young animals, as they may adversely affect the gut health of young broiler chickens [3]. Therefore, there is growing interest in enhancing the nutritional quality of SBM while minimizing the negative impacts of its ANF using effective and economical strategies, such as fermentation [4–6].

For decades, fermentation has emerged as one of the most cost-efficient methods to enhance the protein quality of SBM. Many studies have investigated the use of diverse microbial strains as inoculants for producing fermented SBM (FSBM). Specific microbial strains, such as *Bacillus subtilis* (BS) and *Aspergillus oryzae* (AO), can degrade the ANF content of SBM, thereby increasing the bioavailability of AAs and other essential nutrients for the animals. Studies have demonstrated that different strains of BS could effectively reduce the levels of ANF such as protease inhibitors, β-conglycinin, phytic acid, glycinin, and fiber content while increasing crude protein (CP) level of SBM [3,7]. Fermenting SBM using AO was also reported to increase AA content and bioactive compounds’ levels of SBM [8,9]. BS is well-known as a producer of extracellular enzymes, such as protease and lipase, that can break down the protein matrix of SBM into digestible peptides and AAs. Similarly, AO facilitates the degradation of ANF and the release of bioactive compounds [8].

Numerous *in vivo* studies have demonstrated that partially replacing SBM with FSBM positively modulated the composition of the intestinal microbiome in broiler chickens — a key determinant of gut health and nutrient metabolism [10,11]. The supplementation of FSBM prepared using various inoculants, mostly BS, also enhanced the gut integrity, antioxidant capacity, and immune status of broiler chickens and laying hens [12,13]. A recent meta-analysis of 16 studies revealed that FSBM using a single inoculant of BS or AO had a superior effect on improving the performance of broiler chickens [14].

While previous studies have explored the individual effects of microbial fermentation on SBM, the combination of BS and AO remains underexplored. There is no or very little knowledge on the effects of dual inoculant of BS and AO along with the two-step mechanical and fermentation process of SBM on broiler chickens’ production and intestinal microflora. Our laboratory-scale study has demonstrated that the FSBM using mechanical pretreatment and dual inoculants had higher improvement effects on AA composition and antinutritional profile [15,16]. Therefore, we hypothesized that incorporating FSBM using BS and AO could modulate the intestinal microbiota composition, thereby enhancing the production performance of broiler chickens. To verify this hypothesis, this study was carried out to examine the effect of FSBM on growth performance, nutrient digestibility, and cecal microbial composition of broiler chickens.

## MATERIALS AND METHODS

### Production of fermented soybean meal

The primary objective of this study was to examine the compensatory effects of totally replacing SBM with FSBM on the production performance and cecal microbial compositions of broiler chickens raised in tropical environments. The FSBM was produced through two-step mechanical and solid-state fermentation (SSF) with detailed procedures described in our previous publication [15]. The SBM used in this study contained 48% CP and 91% dry matter (DM) and was sourced from a commercial poultry shop in Surakarta, Indonesia. The material was originally imported from Brazil. A hammer mill (Thomas Model 4 Wiley Mill 2012; Thomas Scientific) was used to grind the SBM to pass through a 1 mm screen. Then, the ground SBM (10 kg at a time) was sterilized for 15 min in an autoclave at 121°C and cooled down before fermentation. For the fermentation, BS was prepared in Luria broth (LB) liquid medium (10 g tryptone, 5 g yeast extract, 5 g NaCl, and 20 g agar) at 37°C for 24 hours to prepare BS inoculum with a cell density of 5×10^9^ CFU/g. AO was prepared on potato dextrose agar (PT Merck Chemicals and Life Sciences) for 7 days at room temperature (~25°C) to allow spore formation. The final spore suspension had 5×10^7^ CFU/g.

The SSF to produce FSBM was conducted in two sequential steps at a large scale to produce 100 kg of FSBM for each group. Briefly, a one-step fermentation was performed on SBM inoculated with either BS or AO. For this, an equal volume of distilled water (50:50 w/v) pre-mixed with inoculant was added onto the sterilized SBM with a final cell density of BS or AO of at least 1×10^8^ CFU/g or 1×10^6^ CFU/g, respectively, during fermentation. The inoculated SBM was then incubated at 25°C for 24 hours for BS and 72 hours for AE, respectively. A two-step fermentation was performed using the above procedure to produce the BS-AE. The fermentation was done under static aerobic conditions. Upon completion, the fermented products (10 kg at a time) underwent mechanical processing via steam conditioning at 121°C for 15 min in an autoclave.

### Experimental design and management of broiler chickens

A total of 160 one-day-old chicks of sex-mixed (male and female sex ratio: 50/50) Cobb 500 broiler chickens with an average body weight (BW) of 38.5 grams were purchased from the commercial breeding farm (Cheil Jedang Superfeed Indonesia, Wonogiri Farm) and were enrolled in this experiment. The DOC were randomly assigned according to the initial BW (day 14) into four dietary groups, distributed into pens, whereas each treatment group was replicated four times (pens as an experimental unit) with 10 birds in each pen. The treatment group consisted of control (broilers received the basal diet; CON) and treatment groups in which the SBM of basal diet was replaced by FSBM produced using AO group, BS group, and the combination of AO and BS (AO+BS group). The ingredients and nutrient composition of the experimental diets are presented in Table 1.

The birds received a commercial starter diet until day 14 and the experimental diets according to their treatment groups from day 15–35. The bird management followed the Cobb 500 broilers management guide [17]. The birds were raised in a conventional management system with an open-house system and natural ventilation to mimic the common practice of conventional broiler chicken farms in Indonesia. The lighting was maintained 24 hours for the first 7 days and then reduced to 20 hours until day 35. The temperature was maintained at 32°C during brooding (48 hours) and then gradually reduced to 31°C for the next 48 hours. Thereafter, the temperature was kept at around 30°C and 85% relative humidity. The birds received ad libitum fresh water through the experiment and feed was provided ad libitum as well. Vaccination programs were implemented, and all birds received Newcastle Disease (ND) on day 4, avian influenza (AI) on day 10, and Gumboro on day 14.

### Growth performance measurement

The BW and feed intake were recorded at the end of the experiment on day 35. The mortality, if any, was recorded daily. The BW gain (BWG) was calculated as the difference between final BW on day 35 with the initial BW on day 14 in which the experiment was commenced. The FCR was calculated as: FI/BWG, corrected by mortality. At the end of the experiment (day 35), three birds from each group with BW close to the average of the group BW were euthanized after being fasted for 12 hours. After evisceration, carcass and organ including spleen, heart, lymph, liver, abdominal fat, intestine, and total inner organ were weighed. The organ weight was reported as the relative weight to the BW (% BW). In addition, approximately 5 grams of cecal content from CON and AO+BS groups were collected. The decision to use of AO+BS treatment was based on the highest production performance of the birds compared to AO and BS alone and was due to the limited financial budget. The cecal contents were temporarily stored in liquid nitrogen. Then, the cecal content was immediately sent to PT Genetics Science Indonesia for 16S rDNA isolation and sequencing.

### Digestibility study

Two birds from each pen with representative BW from each group were selected for *in vivo* digestibility study (day 30). The birds were moved into an individual cage and received the experimental diets over a five-days of total collection period during which the excreta were collected. The collected excreta were pooled and homogenized for each pen, and sundried. Although the sun-drying might not be ideal method to dry excreta sample, previous studies indicated that sun-drying had little effect on DM, organic matter (OM), and CP content of various biological samples [18,19]. The samples were milled through a 0.5 mm mesh screen and subsequently analyzed for proximate composition, including CP content (Kjeldahl method), DM, ether extract (EE), crude fiber (CF), and crude ash according to the AOAC International [20]. The nutrient intake was calculated by multiplying the nutrient content of the diets with feed intake during the digestibility trial and the amount of excreted nutrient was calculated by multiplying the nutrient content of the excreta with the amount of the extreta. Thereafter, the apparent nutrient digestibility was calculated as:


(1) $$\begin{array}{l} \text{Apparent nutrient digestibility }(\%)=\\ \frac{\text{Nutrient intake }(\text{g})-\text{Nutrient excreted }(\text{g})}{\text{Nutrient intake }(\text{g})}\times 100\%. \end{array}$$

### Microbial DNA isolation and 16s rDNA sequencing

The isolation and sequencing of 16s rDNA of cecal microbial composition was done using V3-V4 hypervariable region through next-generation sequencing (NGS) technology on the Illumina MiSeq 2500 platform. For this analysis, total DNA was extracted from three biological samples from the CON and AO+BS groups, respectively, using Quick-DNA Microbe Kit (#D3020; Zymo Research) following the protocol instructions. The DNA concentration was diluted to a similar concentration, and unique primer combinations were added to each sample. The DNA samples were amplified using PCR and the PCR products were verified via 1% agarose gel electrophoresis to confirm the base length. Following this step, a normalization step was performed, and the samples were pooled for library preparation. The pooled sample underwent library construction and quality control, and the final concentrations were determined. The forward 341F (5’-CCTAC GGGNGGCWGCAG-3’) and reverse 785R (3’-GACTACH VGGGTATCTAATCC-5’) primers were used from the Illumina kit and the samples were loaded onto the Illumina MiSeq 2500 platform (Illumina).

### Microbiome data analysis and functional prediction analysis

The demultiplexed FASTQ files received from PT Genetics Science Indonesia were independently processed through the QIIME2 pipeline (ver. 2024.2) [21]. Briefly, the FASTQ files were imported into QIIME2 via the Casava 1.8 paired-end protocol, in which the denoising and quality filtering were performed using the DADA2 algorithm embedded in QIIME2 [22]. Taxonomic classification was performed by assigning the sequences to the 99% V3-V4 pre-trained SILVA classifier (ver. 138) [23,24].

Analysis of alpha and beta diversity was conducted within the QIIME2 tool. Multiple alpha-diversity parameters, including Chao1, ACE, Shannon, and Simpson, were used, while beta-diversity analysis used Bray-Curtis dissimilarity and ANOSIM statistical tests. The beta-diversity was visualized in principal coordinate analysis (PCoA). Differential abundance analysis was performed using DESeq2 with FDR-corrected p-value [25]. Raw count data were rarefied to the lowest read count (36,051 reads) to ensure robustness. Further normalization was applied using the centered log-ratio transformation. Linear discriminant analysis effect size (LEfSe) analysis was carried out to identify microbial features or biomarkers affected by the treatments. The Mann-Whitney test was applied for group comparisons, accounting for the compositional and noisy nature of microbiome data. In addition, functional KEGG pathway predictions were generated using the PICRUSt2 pipeline, which converts the taxa into KEGG ortholog (KO ID). The dataset of KO abundance was submitted to the STAMP software profile [26] to identify the significantly influenced pathways.

### Statistical analysis

Data on production performance parameters were subjected to analysis of variance using PROC MIXED of SAS (SAS ver. 9.4; SAS Institute) after the data normality was tested and confirmed. For the analysis, dietary treatments were set as the fixed effect, and the pens were set as the random effect. Initial BW, which was recorded on day 15, was used as a covariate in the model. The significant difference between experimental groups was determined using Tukey multiple comparison test at p<0.05. Data were presented as the mean values with pooled standard error of the means (SEM).

## RESULTS

### Production performance

The results of production performance measurement (Table 2) indicated that the birds fed AO had similar final BW with the CON group while BS tended (p = 0.063) to improve final BW. Birds fed AO+BS diet resulted in higher (p = 0.003) final BW than CON or AO. Similarly, birds fed FSBM prepared under either BS or AO+BS consumed higher feed intake (p<0.01), while AO did not result in a difference than the CON group. No difference was found in the FCR, regardless of the inoculant used for fermentation. Lower (p<0.01) heart, liver, abdominal fat, and total inner organs were found in birds in response to the AO+BS diet, but relative carcass weight did not differ from CON.

### Nutrient digestibility

Table 3 presents the nutrient digestibility and digestive enzyme activity of broiler chickens fed FSBM diets. Treatments with BS or AO+BS promoted greater DM (p = 0.032), OM (p = 0.016), and CP (p = 0.044) digestibility compared to CON. In contrast, higher OM digestibility was observed (p<0.01) on AO treatment compared to CON, but the digestibility of DM and CP was unaffected.

### Cecal metagenomic profile

**Figure 1:**
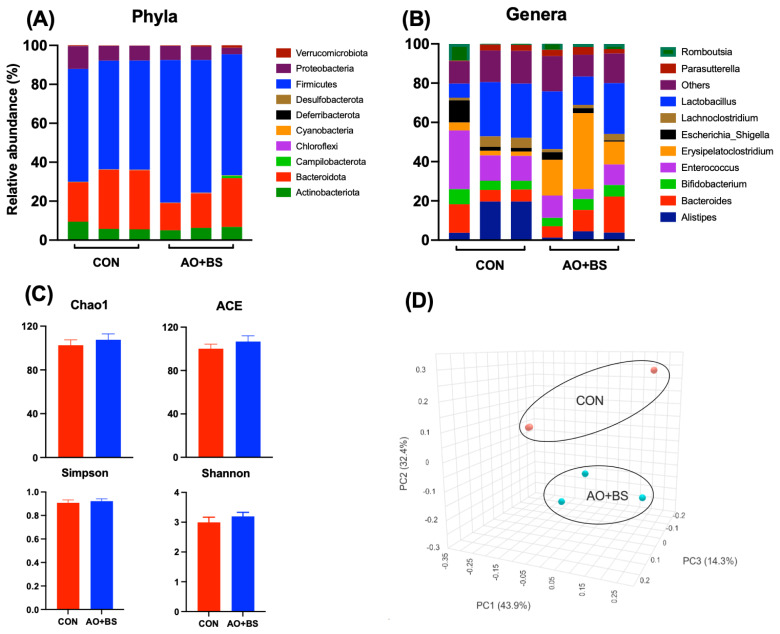
The effect of fermented soybean meal produced using a combination of *Aspergillus oryzae* and *Bacillus subtilis* (AO+BS) or control (CON) diet on cecal microbiota of broiler chickens. (A, B) are the relative abundance of microbial Phyla and Genera, respectively. (C) is α-diversity matrix representing the richness and evenness of the microbes while (D) is principal coordinate analysis (PCoA) of β-diversity index representing bacterial community matrix between CON vs. AO+BS groups.

At the phylum level, differential abundance analysis revealed that AO+BS treatment resulted in a higher proportion of Firmicutes than the CON group. At the same time, the other phyla were not altered, but the relative abundance of Bacteroidota tended to be lower in the AO+BS vs. CON group. At the genus level, *Bacteroides* and *Erysipelatoclostridium* were enriched in the AO+BS group, while *Alistipes* were enriched in the CON group (Figure 1). However, there was no difference in alpha-diversity and beta-diversity metrics between the two groups.

### Functional pathway analysis

**Figure 2:**
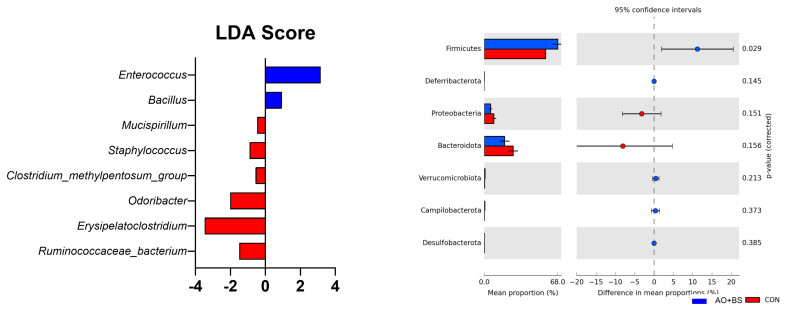
Linear discriminant analysis (LDA) score highlighting microbial taxa that were affected by fermented soybean meal prepared with *Aspergillus oryzae* and *Bacillus subtilis* (AO+BS) compared to control diet (CON). The right quadrant is the statistical result of differential abundance analysis of microbial Phyla.

**Figure 3:**
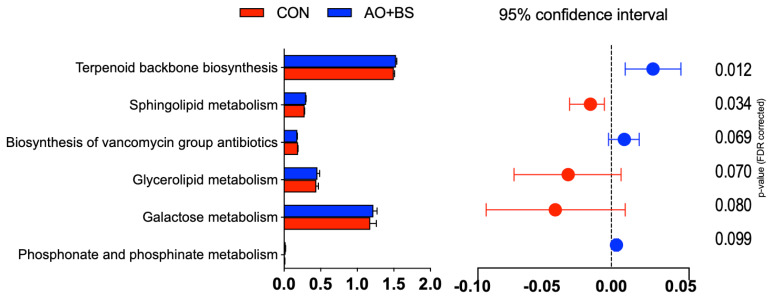
KEGG pathways prediction analysis based (relative abundance with 95% confidence interval of differential relative) on microbial taxa of broiler chickens fed control (CON) or fermented soybean meal prepared using *Aspergillus oryzae*+*Bacillus subtilis* (AO+BS) diet.

The LEfSe analysis identified several microbial taxa that were more abundant in the AO+BS compared to the CON groups (Figure 2). Broiler chickens fed the AO+BS diet had a greater abundance of *Enterococcus* and *Bacillus* as indicated by the positive LDA scores. On the other hand, several genera such as *Erysipelatoclostridium*, *Odoribacter*, *Ruminococcaceae bacterium*, *Staphylococcus*, and *Clostridium methylpentosum* group were less abundant in the CON group. The prediction of KEGG functional pathways showed only a few differences, including the enrichment of Terpenoid backbone biosynthesis in the AO+BS group (p = 0.012) and downregulation of Sphingolipid metabolism in the CON group (p = 0.034; Figure 3). Several KEGG pathways showed a statistical tendency such as downregulation of glycerolipid (p = 0.07) and galactose metabolism (p = 0.08) in the CON group and upregulation of phosphonate and phosphinate metabolism (p = 0.099).

## DISCUSSION

Data of the production performance parameters in this study demonstrated that feeding FSBM produced using BS and AO as inoculants resulted in the highest BWG compared to birds fed FSBM produced using a single inoculum (AO or BS alone) and CON. Several factors could explain the favorable effects of AO+BS treatment, but the higher nutrient intake and the lower antinutritional content (ANF) intake might be the primary driving factors. As demonstrated in our previous experiment [15], positive alterations in AAs, ANF, and antioxidant potential of FSBM prepared using AO+BS could favorably enhance nutrient digestion, promote better gut health, improve protein turnover, and potentially enhance the antioxidant and mucosal immune status of the birds. A previous study supported our finding whereas SSF of SBM increased CP content by 8.4% and essential AAs content by 3.5% while concomitantly decreased phytic acid content by 53.6% [15]. The improved SBM nutritional quality could be the major factor contributing to higher BWG because better nutrient quality would promote its utilization by the animals. However, FCR was unaffected by FSBM treatment, mainly due to the higher feed intake of birds fed FSBM (Table 2). The higher feed intake was particularly found in the BS and AO+BS groups while the feed intake of the AO group did not differ compared to the CON group. This suggests that BS could be the driving factor of higher feed intake, which might be attributed to the improvement of palatability. The reduced ANF content and enriched bioactive peptides might lead to appetite-stimulating effects. Those factors could lead to faster passage rate due to multiple enzymes produced by BS as well as might promote positive microbial composition in the intestinal tract [2,7]. Improved microbial profile in broilers was reported to produce greater short-chain fatty acids (SCFA), which are known to have feed intake-stimulating effect via gut-brain axis signaling [5,7,13]. Although similar results of higher feed intake were reported in studies using FSBM on broiler chickens [27,28], other studies also reported no effect on feed intake [29,30], which might be due to differences in microbial strain used for fermentation and environmental conditions (tropical hot climate). Nonetheless, this result warrants further investigations using a larger scale trial.

It is well known that fermentation increases nutrient digestibility by reducing fiber content and increasing protein, AAs, and lipid contents of SBM [12,31,32]. During fermentation, BS releases extracellular enzymes that facilitate fiber matrix breakdown that would subsequently enhance carbohydrate and ANF digestion, thereby leading to lower pH, higher organic acid production, and elimination of various ANF. Qi et al [3] studied the ability of various enzymes produced by BS and demonstrated that the bacterial strain had a remarkable ability to digest ANF including glycinin, β-conglycinin, xylan, corn starch, CMC, and allergenic proteins due to multiple enzymes produced by BS such as xylanase, amylase, cellulase, and protease. Similar results of reduced ANF and increased AAs and peptide contents were reported in SSF of SBM using various BS strains [31–35]. Previous studies that examined AO for fermenting SBM also reported increased CP, EE, Ca, and P digestibility [12] as well as decreased protease inhibitors (trypsin and chymotrypsin inhibitors activities) by 21%–23% [8]. In addition, the latter study also observed higher phenolic content in the materials fermented by AO, similar to our finding [15]. The above well-established mechanism would ultimately contribute to increase nutrient digestibility in broiler chickens.

In addition, it was suggested that FSBM increased concentration of digestible peptides, which could promote higher protein utilization efficiency by broiler chickens [36,37]. Previous experiments also reported the enhanced gut physiology, as indicated by improved epithelial cell structure, higher villi volume and crypt depth, lower pH of the small intestine, and higher SCFA production in broiler chickens fed FSBM [11,35,38]. Incorporating 6% FSBM to replace conventional SBM was reported to suppress total fungi and *Coliforms* in the jejunum [39] and ameliorated *Salmonella* Typhimurium infection, resulting in greater production performance of broiler chickens [40]. The higher abundance of lactic acid bacteria might have increased the production of organic acids, benefiting the gut environment. It is well established that organic acids exert antimicrobial activity due to their bacteriostatic and bactericidal activities [41–43]. Numerous publications have demonstrated that the use of single or multiple strains of inoculum to produce FSBM resulted in greater production performance of broiler chickens by regulating mucosal immunity and gut microbial composition [10,13,44].

Regarding the positive alterations of cecal microbial composition, the higher abundance of *Enterococcus* may indicate gut health improvement of the birds, as some *Enterococcus* species are known as probiotics. In the last few years, a number of studies have reported various *Enterococcus* species, such as *Enterococcus faecium* L3, *Enterococcus Faecium* NCIMB 11181, and *Enterococcus Faecalis-1* as potential probiotics for broiler chickens [45–48]. In addition, *Enterococcus* also exhibited ameliorating effects on pathogen infections in broiler chickens such as *E. coli* [46,49] and *Salmonella* Typhimurium [48]. These promising effects are particularly desirable for broiler chickens raised in tropical environments, in which broiler chickens often experience higher challenges of pathogen invasion and other stressors that impair their production potential. Similarly, the enrichment of *the Bacillus* genus is likely a direct consequence of its utilization as an inoculant for FSBM production, which supports its colonization. The advantages of *Bacillus* species as probiotics have been reviewed [14,50], highlighting its promising use as a feed additive for broiler chickens. Thus, the higher abundance of *Bacillus* might also explain greater nutrient digestibility to produce SCFA in the hindgut of broilers, which contributes to providing higher energy supply for the birds. The positive modulatory effect of the cecal microbiota in this study were also reported in previous studies examining the partial replacement of SBM with FSBM in broiler chickens [7,10–12].

Taxa such as *Erysipelatoclostridium*, *Odoribacter*, *Ruminococcaceae bacterium*, *Staphylococcus*, and the *Clostridium methylpentosum group* were enriched in the CON group. The greater abundance of these taxa indicated an unbalanced or less beneficial microbial community in the CON group. For instance, several *Erysipelatoclostridium* and *Staphylococcus* species are often classified as opportunistic microbial pathogens [51–53], and their enrichment could be detrimental for the hindgut section environment. The observed effect highlighted the positive impact of the AO+BS treatment on the cecal microbiota whereas the abundance of bacteria such as *Enterococcus* and *Bacillus* increased while higher abundance of pathogenic taxa was observed on the CON group. This shift in cecal microbial composition is reflected in the higher BW of the AO+BS group. Additionally, the decrease in taxa such as *Erysipelatoclostridium* and *Odoribacter* suggests that the AO+BS treatment reduced the colonization of microbial pathogens that could lead to inflammation or inefficiencies. These findings align with previous research demonstrating the beneficial effects of probiotic supplementation on microbial balance and broiler health [54,55].

Analysis of KEGG pathway prediction in this study showed minimal effect on metabolic potential of cecal microbiota. Interestingly, the AO+BS diet enriched the terpenoid backbone biosynthesis pathway, which is essential for isoprenoids production as precursor of antioxidant biomolecule production [56]. The enrichment of this pathway was also associated with enhanced membrane cell integrity and immune system [57], suggesting an improved immune system and antioxidant status of broiler chickens fed AO+BS diet. In contrast, broiler chickens fed CON diet exhibited downregulation of sphingolipid metabolism. This pathway plays essential roles in cell signaling, apoptosis, and maintaining intestinal barrier integrity [58]. A reduction in this pathway could reflect a less favorable microbial activity profile in the CON group, possibly contributing to impaired gut homeostasis. Trends toward lower glycerolipid and galactose metabolism in the CON group further support this pattern, as both pathways are related to energy utilization and carbohydrate metabolism, which are essential for supporting rapid growth in broilers. Thus, although minimal, these functional predictions, suggest that AO+BS diet not only altered microbial composition but also may have enhanced specific metabolic pathways that support host antioxidant capacity, nutrient utilization, and gut health.

Despite the promising findings on the beneficial modulatory effects of the cecal microbiota in response to AO+BS diet, the limited or minimal sample size (n = 3 biological replicates) should be noted as the major limitation. The sequencing depth and resolution were restricted to genus-level interpretation, which may not fully capture functional dynamics or strain-specific roles of the identified taxa. Moreover, the KEGG predicted pathways were not validated with other methods such as transcriptomic or RT-qPCR method, which would provide deeper insights into microbial functions, metabolic pathways, and their direct interactions with host physiology. Future studies should consider a larger and representative sample size from larger commercial studies. In addition, performing integrated multi-omics approaches, including metatranscriptomics and metabolomics would be better to elucidate the mechanisms by which FSBM-driven microbial shifts contribute to nutrient utilization, immune modulation, and production performance in broiler chickens.

## CONCLUSION

Incorporating FSBM produced using BS and AO resulted in greater effects than a single inoculant to improve the production performance of broiler chickens raised in tropical environments. The positively altered cecal microbial profile of broilers fed an AO+BS diet could partly explain the improved broiler production, supporting its use as a potential dietary strategy. Future studies could further investigate the functional pathways associated with these microbial shifts to better understand the mechanisms behind the observed benefits.

## Figures and Tables

**Table 1:** Ingredients and nutrient composition of basal and experimental diets

Ingredients (%)	Diet composition				

	Day 1–14	Day 15–35			
	
	Starter^1)^	CON	AO	BS	AO+BS
Maize		42.18	43.50	43.46	44.42
Rice bran		15.00	15.00	15.00	15.00
Palm oil		0.25	0.25	0.25	0.25
Soybean meal (SBM)		20.00	0.00	0.00	0.00
Fermented soybean meal (FSBM)		0.00	20.00	20.00	20.00
Full fat soyabean		17.83	16.88	16.91	16.22
DL-methionine		0.24	0.24	0.24	0.24
L-lysine		0.39	0.26	0.32	0.28
Vitamins premix		1.73	1.49	1.43	1.20
Minerals premix		2.00	2.00	2.00	2.00
Sodium salt		0.39	0.39	0.39	0.39
Total		100	100	100	100
	Nutrient composition				
Metabolizable energy (kcal/kg)	3,051	2,950	2,950	2,950	2,950
Crude protein (%)	21.21	20.82	20.50	20.50	20.76
Crude fiber (%)	3.28	2.22	2.20	2.20	2.18
Ether extract (%)	4.08	7.40	7.22	7.26	2.26
Methionine (%)	0.56	0.49	0.49	0.49	0.49
Lysine (%)	1.10	1.23	1.31	1.36	1.38

The treatment group consisted of control (broilers received basal diet; CON) and treatment groups in which the SBM of basal diet was replaced by FSBM produced using *Aspergillus oryzae* (AO), *Bacillus subtilis* (BS), and the combination between AE and BS (AO+BS).

1) Starter diet was purchased commercially in a fine crumble form (BR1; PT Japfa Comfeed Indonesia Tbk).

**Table 2:** Production performance of broiler chickens fed fermented soybean meal (SBM) processed under different microbial cultures

Items	Experimental diets^1)^				SEM	p-value

	CON	AO	BS	AO+BS		
Production performance						
BWG (g)	1,361^b^	1,368^b^	1,480^ab^	1,530^a^	35.4	0.003
FI (g)	2,220 ^c^	2,279^c^	2,506^ab^	2,715^a^	55.3	<0.001
FCR	1.63	1.67	1.70	1.78	0.046	0.158
Relative organ weight (% BW)						
Spleen	0.13	0.10	0.12	0.09	0.015	0.449
Heart	0.72^a^	0.66^b^	0.71^a^	0.60^b^	0.030	0.003
Lymph	0.13	0.17	0.17	0.12	0.021	0.208
Carcass	63.67^a^	62.41^b^	65.36^a^	64.70^a^	0.545	0.006
Liver	2.07^b^	2.29^a^	2.33^a^	1.71^c^	0.084	<0.001
Abdominal fat	0.51^b^	0.99^a^	1.03^a^	0.26^c^	0.164	0.002
Intestine	7.56^b^	8.25^a^	8.13^a^	6.47^c^	0.240	<0.001
Inner organ	11.41^b^	13.13^a^	14.20^a^	8.53^c^	0.291	<0.001

1) The treatment group consisted of control (broilers received basal diet; CON) and treatment groups in which the SBM of basal diet was replaced by fermented SBM produced using *Aspergillus oryzae* (AO), *Bacillus subtilis* (BS), and the combination between AO and BS (AO+BS).

a–c Different superscripts within the same row indicate statistical significance at p<0.05.

SEM, standard error of the means; BWG, body weight gain; FI, feed intake; FCR, feed conversion ratio; BW, body weight.

**Table 3:** Nutrient digestibility of broiler chickens fed fermented soybean meal (SBM)

Parameters	Experimental diets^1)^				SEM	p-value

	CON	AO	BS	AO+BS		
Digestibility						
DMD	59.77^b^	65.01^ab^	67.17^a^	67.91^a^	2.069	0.032
OMD	65.73^c^	70.71^b^	72.40^a^	73.45^a^	1.468	0.016
CPD	51.95^b^	51.79^b^	54.91^ab^	59.83^a^	2.312	0.044

1) The treatment group consisted of control (broilers received basal diet; CON) and treatment groups in which the SBM of basal diet was replaced by fermented SBM produced using *Aspergillus oryzae* (AO), *Bacillus subtilis* (BS), and the combination between AO and BS (AO+BS).

a–c Different superscripts within the same row indicate statistical significance at p<0.05.

SEM, standard error of the means; DMD, dry matter digestibility; OMD, organic matter digestibility; CPD, crude protein digestibility.

## Data Availability

Upon reasonable request, the datasets of this study can be available from the corresponding author.
